# Treating iron deficiency in patients with gastrointestinal disease: Risk of re-attendance in secondary care

**DOI:** 10.1371/journal.pone.0189952

**Published:** 2017-12-15

**Authors:** Susannah Tomkins, Callum Chapman, Melissa Myland, Rachel Tham, Rachael de Nobrega, Brinley Jackson, Satish Keshav

**Affiliations:** 1 NEMEA Centre of Excellence for Retrospective Studies, IQVIA, London, United Kingdom; 2 Chelsea and Westminster Hospital, London, United Kingdom; 3 Vifor Pharma UK Ltd, Surrey, United Kingdom; 4 Gastroenterology Unit, John Radcliffe Hospital, Oxford, United Kingdom; Pennsylvania State University College of Medicine, UNITED STATES

## Abstract

**Background:**

Patients with gastrointestinal disease may have comorbid iron deficiency anaemia (IDA) and an increased risk of hospitalisation and re-attendance in hospital. The purpose of this study was to determine if oral and intravenous (IV) treatment of IDA in patients with gastrointestinal disease attending hospital were associated with differential rates of subsequent re-attendance.

**Methods and findings:**

Data from the Clinical Practice Research Datalink (primary care) and Hospital Treatment Insights (secondary care) databases in England were used to conduct this retrospective cohort study. Patients with a coded gastrointestinal disease and IDA who attended hospital (inpatient or outpatient) and were dispensed oral or IV iron between 01/01/2010-31/10/2013 were included. Elective and emergency re-attendances in secondary care within 30 days of the initial attendance were determined. Demographics, medical diagnoses and treatments were extracted. Re-attendance rates following oral or IV iron were compared using chi-square tests and a step-wise logistic regression model to adjust for confounders. 2,844 patients contributed 6,294 initial attendances; 80% of patients received oral iron, 14% received intravenous iron, and 6% received both. Of initial attendances recording oral iron, 77% resulted in re-attendance in hospital, compared to 34% of those recording IV iron (unadjusted odds ratio [OR]: 0.16; adjusted OR: 0.52 [95% CI: 0.44–0.61]). Initial attendances using IV treatment were more likely to result in elective re-attendance (84%) than those recording oral treatment (43%) (p<0.001). Median length of stay in hospital tended to be shorter for patients using IV iron (1.4 days; interquartile range 0.5–3.6 days; oral iron: 5.1 days; interquartile range: 2.2–9.6 days).

**Conclusions:**

Patients with gastrointestinal disease and IDA who received IV iron were less likely to re-attend hospital, more likely to re-attend electively, and tended to have a shorter length of stay in hospital. The mode of IDA treatment could have a real-world impact on healthcare utilisation.

## Background

Iron deficiency (ID) and iron deficiency anaemia (IDA) are common disorders frequently associated with gastrointestinal (GI) disease [1], partly due to blood loss from the GI tract [2] and impaired absorption of iron secondary to GI disorders [3]. IDA is prevalent in 2–5% of adult men and post-menopausal women in the developed world [4]. GI conditions frequently associated with ID and IDA include upper and lower gastrointestinal haemorrhage, peptic ulcer, and inflammatory bowel disease (IBD) [1,4]. Patients with anaemia, regardless of aetiology, are more likely to attend hospital, and have increased mortality [5–7]. Repeated attendance in secondary care is associated with higher costs [8] and re-attendance after an initial visit is widely used as a metric to assess quality of care in hospitals [9,10]. It is not known if co-morbid anaemia in GI disease is associated with higher rates of re-attendance [1]. Concomitant GI disease may contribute to ID and IDA in patients with other primary diagnoses including congestive cardiac failure, chronic kidney disease, and pulmonary disease [1,11]. Thus, ID and IDA may influence re-attendance in patients regardless of whether GI disease is a primary diagnosis or a comorbidity.

Although previous studies have demonstrated that IDA contributes to an increased risk of mortality and re-attendance [11,12], there are limited data on the impact of specific treatments. The main therapeutic options for treating ID are oral supplementation with a variety of ferrous compounds or intravenous (IV) supplementation. IV iron is gaining popularity, partly because modern formulations do not incur the same risk of severe hypersensitivity reactions historically associated with high molecular weight iron-dextran compounds [13]. Modern IV preparations also offer the potential for more rapid iron replacement compared to older preparations and oral treatment [3,14] and may thus be particularly efficacious in patients with GI disorders where intestinal absorption of iron is impaired [3]. As IV iron is generally better tolerated than oral iron, it may be more effective [15] with a lower incidence of side effects [16]. Therefore, this study endeavoured to determine if oral and IV treatment for IDA in patients with GI disease were associated with differential rates of subsequent re-attendance in secondary care.

## Methods

### Study design

**Fig 1:**
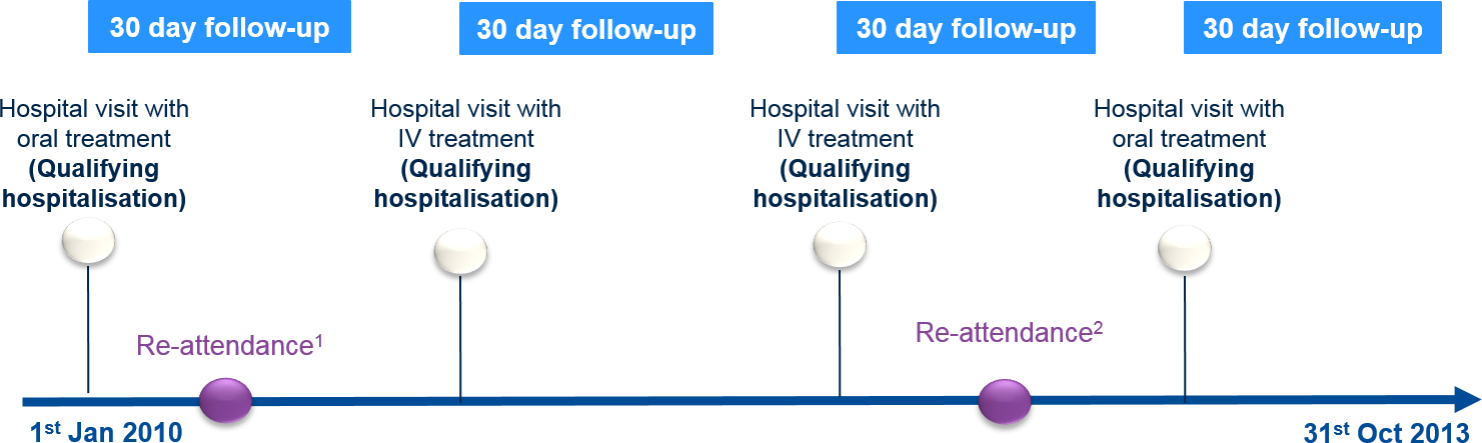
Schematic overview of study design. Following the start of the study period, 1^st^ January 2010, 4 theoretical qualifying events are shown. Within the 30 day follow up period, a re-attendance in hospital is observed for 1) an episode with oral treatment and 2) an episode with IV treatment. Each initial attendance enters into either the oral or IV group and a subsequent re-attendance may or may not occur during the 30 day follow-up period.

This was a retrospective cohort study comparing patients with a GI diagnosis treated with oral and/or IV iron therapy in secondary care. A qualifying attendance (initial attendance) was defined as an episode in hospital (inpatient or outpatient) associated with dispensing of either oral or IV iron; a subsequent re-attendance in secondary care was defined as an attendance within 30 days of the end of the initial attendance. The index date was the final day of the initial episode. Thirty days was chosen because in the UK National Health Service (NHS), re-attendance within 30 days is monitored as a proxy measure of effective secondary care, whereas re-attendance after 30 days is considered a separate event [17]. The analysis was based on discrete episodes rather than individual patients and any patient could have contributed multiple initial attendances in the study time period (01/01/2010-31/10/2013) (Fig 1).

### Data sources

The study used routinely collected data from the Hospital Treatment Insights (HTI)-linked to the Clinical Practice Research Datalink (CPRD) database from 01/01/2010-31/10/2013. HTI contains secondary care data from 44 hospital trusts in England with 4.5 million patients within the dataset, accounting for approximately 25% of patients in England [18]. Hospital-dispensed prescriptions originated from the Hospital Pharmacy Audit dataset (QuintilesIMS, UK) linked to patient-level inpatient/outpatient information as part of Hospital Episode Statistics data curated by NHS Digital [19]. CPRD also contains data on general practice consultations, prescriptions, diagnoses, and laboratory tests [20]. Approximately 200,000 patients are available for study in the linked CPRD-HTI dataset [21].

The prescription exposures of interest were oral and IV iron recorded within HTI. IV iron included CosmoFer^®^ (low molecular weight iron dextran), Venofer^®^ (iron sucrose), Monofer^®^ (iron isomaltoside 1000), Ferinject^®^ (ferric carboxymaltose), and Feraheme^®^ (ferumoxytol). Oral forms included generic ferrous sulphate, ferrous gluconate, ferrous fumarate, ferrous glycine sulphate, iron protein succinylate, and iron polysaccharide.

### Patient inclusion and exclusion criteria

Patients who were ≥18 years old at index date with any International Classification of Diseases (ICD)-10 code for IDA (chapter D50) were included. Additionally, patients were required to have an ICD-10 code for a GI disease (K-20-31; K50-52; K55-K59, K63; K90-K93). Patients were excluded if they only recorded anaemia for reasons other than ID according to the following ICD-10 codes: D55-59; D51-53; D61-64; O90.81; D46.

### Statistical methods

Descriptive analysis was performed using means and medians for continuous variables (e.g., number of re-attendances, length of stay [LOS]). Categorical variables such as age ranges were represented as proportions. Re-attendances post-treatment were described as counts at the episode-level (number of re-attendances per treatment) and compared between oral and IV treatments to compute the odds ratio of re-attendance with 95% confidence intervals (CIs). A logistic regression model was used to compare mean number of re-attendances between therapies, controlling for confounders. As a unique episode for this analysis was defined by a prescription of IV or oral iron, these categories are non-overlapping; however, individual patients could have recorded IV, oral, or both treatments in patient-level analyses.

Visit type (elective or emergency) was coded for both initial attendance and re-attendance to address bias in the type of re-attendance according to treatment. Chi-squared and p-values were calculated to determine any statistically significant differences.

Potential confounding factors that could affect therapy choice or the risk of re-attendance were extracted for logistical regression modelling. A step-wise regression was used to determine which confounders had a significant effect on the outcome at a 5% significance level. Based on this approach, confounding variables in the final model included all which were entered: age, sex, menorrhagia, pregnancy, pharmacological treatments, elective vs. emergency initial attendance, reduced kidney/liver function, and New York Heart Association Class IV heart failure.

### Ethics

Ethical approval was obtained from ISAC on 2nd October 2014, protocol number 14_164R. Informed consent by subjects was not required.

## Results

Overall, 2,844 patients had initial attendances in secondary care and contributed 6,294 episodes for analysis. Patients recorded a median of 3 (interquartile range [IQR]: 0–7) subsequent re-attendances. IV iron was recorded during the initial attendance in 392 patients (14%), oral iron was recorded for 2,267 patients (80%), and mixed therapy was recorded in 185 patients (7%). At the episode-level, IV therapy was recorded in 1,491 (24%) attendances, while oral treatment was recorded in 4,803 (76%) attendances.

### Demographics

Oral iron was less commonly recorded in younger age groups (<37 years) and was more frequent in older patients, whereas the use of IV iron was more uniform across the age groups. The sex distribution was broadly similar across treatment groups, with a slightly greater proportion of females (Table 1). Oral iron was recorded in a higher proportion of patients with reduced kidney function (32% oral vs. 26% mixed; 24% IV) and heart failure (30% oral vs. 20% mixed; 5% IV). Reduced kidney function and heart failure were the most common comorbidities in both treatment groups.

**Table 1:** Baseline characteristics for patients at initial attendance.

	Total*		IV*		Oral*		Mixed*	
	N	%	N	%	N	%	N	%
**Age**	N = 2,835		N = 389		N = 2,263		N = 183	
18–27	110	3.9	49	12.5	49	2.2	12	6.5
28–37	115	4.0	43	11.0	55	2.4	17	9.2
38–47	176	6.2	58	14.8	101	4.5	17	9.2
48–57	185	6.5	42	10.7	127	5.6	16	8.6
58–67	325	11.4	58	14.8	241	10.6	26	14.1
68–77	594	20.9	72	18.4	488	21.5	34	18.4
78–87	900	31.6	56	14.3	801	35.3	43	23.2
88+	430	15.1	11	2.8	401	17.7	18	9.7
**Sex**								
Female	1700	59.8	242	61.7	1341	59.2	117	63.2
Male	1144	40.2	150	38.3	926	40.8	68	36.8
**Comorbidities (most prevalent)**	N = 2,375		N = 332		N = 1,879		N = 164	
Reduced Kidney Function	721	30.4	79	23.8	600	31.9	42	25.6
Heart Failure	646	27.2	49	14.8	565	30.1	32	19.5
Oncology Patient	362	15.2	39	11.7	306	16.3	17	10.4
Chronic Liver Disease	186	7.8	26	7.8	142	7.6	18	11.0
Menorrhagia	141	5.9	39	11.7	83	4.4	19	11.6
Pregnancy	27	1.1	6	1.8	15	0.8	6	3.7

*Totals reflect the number of patients with available data.

The incidence of GI diagnoses (upper GI disease, gastritis/disorders of the stomach and duodenum, IBD, diverticulitis, functional bowel disorders) were examined across age groups in the study population. All conditions were most prevalent in the 78–87 year-old age group (e.g. 4.4% upper GI disease including reflux and peptic ulcer disease to 9.9% diverticular disease), except for IBD which was most common in 18–27 year-olds (1.1% vs. 0.5% in 78–87 year-olds).

### Odds of re-attendance following iron treatment

Of 4,803 episodes using oral iron, 3,683 (77%) resulted in re-attendance within 30 days of the initial attendance, compared to only 504 (34%) of 1,491 episodes using IV iron. The unadjusted odds ratio (OR) for a re-attendance in hospital within 30 days following IV iron compared with oral iron was 0.16. Logistic regression adjusting for confounders (age, sex, history of anaemia, elective and emergency initial attendance, menorrhagia, pregnancy, concomitant treatment and history of reduced kidney function) attenuated the OR to 0.52 (95% CI 0.44–0.61)(Table 2). Older age, female sex, history of anaemia, initial emergency visits, menorrhagia, and pregnancy were associated with increased odds of re-attendance. When examined by age range, there was a significantly reduced odds of re-attendance following IV treatment amongst 18–27 year olds (0.43 [0.22–0.82]), 68–77 year olds (0.50 [0.33–0.75]), and 78–87 year olds (0.45 [0.32–0.65]). The sample sizes in the other age ranges were small with insignificant findings.

**Table 2:** Odds ratio for re-attendance following IV vs oral iron treatment adjusted for statistically significant confounders.

Explanatory variable	Odds Ratio	95% CI
IV vs. Oral	0.52	0.44–0.61
Age range in HTI (ascending)	1.55	1.50–1.61
Sex (Female vs Male)	1.25	1.09–1.43
History of anaemia	1.28	1.11–1.49
Emergency vs. elective	1.64	1.41–1.91
Menorrhagia	4.23	3.33–5.47
Pregnancy	6.18	3.49–10.97

### Odds of elective versus emergency re-attendance

Visit type (emergency/elective) for both initial attendance and subsequent re-attendance was available for 1,464 attendances (23% of all episodes), 210 of which recorded IV iron and 1,254 of which recorded oral iron. In this subset, for initial attendances recording IV treatment, elective attendances occurred more frequently than emergency attendances (70% elective vs. 30% emergency). Conversely, initial attendances using oral treatment were more likely to be emergency (83% emergency vs. 17% elective).

Table 3 shows the distribution of elective versus emergency initial attendance and re-attendance in each treatment group. Overall, IV iron was less frequently used in emergency hospital attendances initially (IV: 30% initial emergency attendance vs. 83% for oral) and resulted in fewer emergency re-attendances (16% IV vs. 57% oral) (p<0.001). Initial emergency visits using oral iron were more likely to result in subsequent emergency re-attendance (62%) (p<0.001) (Table 3).

**Table 3:** Elective versus emergency re-attendance following initial visit type, by treatment group.

	Re-attendance					
	Elective		Emergency		Total	
	n	%	n	%	n	%
**Initial Visit Treated with Oral Iron**						
**Elective**	135	62%	82	38%	217	17%
**Emergency**	398	38%	639	62%	1037	83%
**Total**	533	43%	721	57%	1254	
**Initial Visit Treated with IV Iron**	** **	** **	** **	** **	** **	** **
**Elective**	136	93%	10	7%	146	70%
**Emergency**	40	63%	24	37%	64	30%
**Total**	176	84%	34	16%	210	

### Hospitalisation details

The median LOS in hospital was 5.1 days (IQR: 2.2–9.6) in the oral treatment group compared with 1.4 days (IQR: 0.5–3.6) in the IV treatment group. The maximum LOS was also substantially shorter in the IV group in comparison with the oral group (34.1 IV vs. 120.2 oral).

For visits where consultant specialty was recorded, 89% prescribed oral treatment and 11% prescribed IV treatment. General medicine (28% of oral prescriptions), geriatric medicine (21%), and gastroenterology (9%) were associated with the largest proportion of oral iron prescriptions, whereas general medicine (23% of IV prescriptions), clinical haematology (18%) and gastroenterology (17%) accounted for most prescriptions of IV iron.

## Discussion

This study suggests a decreased risk of re-attendance in secondary care for patients receiving IV iron in comparison with oral iron for ID/IDA in patients with GI disease. LOS in hospital tended to be shorter when associated with IV therapy, although findings were not statistically significant; nevertheless, maximum LOS was substantially reduced in this treatment group. IV iron therapy was more often associated with subsequent elective re-attendance, while initial attendances recording oral iron were more likely to result in emergency re-attendance. This raises the possibility that oral iron treatment in this setting may be clinically inadequate. As the NHS prioritises reducing emergency hospital admissions [22], IV iron treatment could be recommended more frequently in efforts to improve patient and economic outcomes by reducing the number and length of hospital admissions.

Despite the increasing number of studies demonstrating benefits of IV iron therapy in patients with ID [23,24], a clear consensus on when to use IV or oral iron is lacking [25,26]. Although health economic outcomes were not ascertained in this study, the results are suggestive of potential reductions in secondary care use. Research suggests that IV treatment could be less costly than oral treatment overall despite greater medication costs, partially influenced by reduced length of hospitalisation [27]. Accordingly, treatment with IV iron therapy may improve the efficiency of care by reducing the need for further treatment or hospitalisation based on better haemoglobin response [26,28] and lower incidence of side effects [25,26].

Previous studies have indicated that IDA in GI disease leads to more frequent hospitalisations along with diminished quality of life, lost time at work [29], and mortality [11,12]. Results from this study suggest that treatment with IV iron has the potential to reduce some of the morbidity associated with IDA in GI disease by decreasing the odds of re-attendance and the likelihood of emergency re-attendances. IV iron may also prevent recurrence of anaemia [30].

This study provided some insights into clinical practice: surprisingly, IV iron was recorded less frequently in patients with CKD, heart failure, and those who were elderly. A comparatively greater proportion of younger patients received oral rather than IV iron. The smaller sample size of the IV cohort may have contributed to this finding. Treatments received from ward stock outside the hospital pharmacy would not have been captured and may have resulted in missing data. Additionally, patients may have been receiving other IDA treatments (e.g., erythropoiesis stimulating agent) [31] and older patients may have been overlooked for the more expensive IV therapy [32]. IV iron appears to be underutilised; the NHS should prioritise its dispensation as IV iron may substantially reduce the risk of re-attendance in hospital and length of stay.

This study is limited by its retrospective nature without direct analysis of individual patient records. Consequently, certain potentially important confounding variables such as smoking status and region of residence could not be evaluated. Furthermore, the number of patients treated with IV iron was small, limiting the statistical power of the study. The data were also derived from hospitals in England and may not be generalizable. Selection bias was minimised by including all patients with a history of GI disease and IDA present in the HTI-CPRD database who met the eligibility criteria. Missing data was accounted by for creating a “missing data” code, rather than by imputation as data were unlikely to fulfil the missing at random hypothesis due to differences in data recording influenced by patient characteristics [33]. Bias may have been introduced by conducting analyses on an episode-level rather than at patient-level, whereby more severely affected patients may have repeatedly re-attended hospital. Although it could be assumed that patients treated with IV iron were more likely to have severe disease [3] and bias results towards higher re-attendance and longer LOS, the opposite was observed in our study.

## Conclusion

This retrospective, real-world database study demonstrates substantially reduced odds of re-attendance in hospital for patients with IDA and a GI condition who were treated with IV iron compared to oral iron. Additionally, IV iron treatment was associated with reduced rates of emergency re-attendance and potentially shorter LOS in hospital. The data suggest that further studies should explore the optimal strategy for treating ID/IDA in the secondary care setting.
